# Characterization of Two Mitochondrial Genomes and Gene Expression Analysis Reveal Clues for Variations, Evolution, and Large-Sclerotium Formation in Medical Fungus *Wolfiporia cocos*

**DOI:** 10.3389/fmicb.2020.01804

**Published:** 2020-08-04

**Authors:** Mengting Chen, Naiyao Chen, Ting Wu, Yinbing Bian, Youjin Deng, Zhangyi Xu

**Affiliations:** ^1^Institute of Applied Mycology, College of Plant Science and Technology, Huazhong Agricultural University, Wuhan, China; ^2^Key Laboratory of Agro-Microbial Resource Comprehensive Utilization, Ministry of Agriculture, Huazhong Agricultural University, Wuhan, China; ^3^Center for Genomics and Biotechnology, Haixia Institute of Science and Technology, College of Life Sciences, Fujian Agriculture and Forestry University, Fuzhou, China

**Keywords:** *Wolfiporia cocos*, mitochondrial genome, sclerotial formation, gene rearrangement, tRNA

## Abstract

*Wolfiporia cocos*, a precious mushroom with a long history as an edible food and Asian traditional medicine, remains unclear in the genetic mechanism underlying the formation of large sclerotia. Here, two complete circular mitogenomes (BL16, 135,686 bp and MD-104 SS10, 124,842 bp, respectively) were presented in detail first. The salient features in the mitogenomes of *W. cocos* include an intron in the tRNA (trnQ-UUG^2^), and an obvious gene rearrangement identified between the two mitogenomes from the widely geographically separated *W. cocos* strains. Genome comparison and phylogenetic analyses reveal some variations and evolutional characteristics in *W. cocos*. Whether the mitochondrion is functional in *W. cocos* sclerotium development was investigated by analyzing the mitogenome synteny of 10 sclerotium-forming fungi and mitochondrial gene expression patterns in different *W. cocos* sclerotium-developmental stages. Three common homologous genes identified across ten sclerotium-forming fungi were also found to exhibit significant differential expression levels during *W. cocos* sclerotium development. Most of the mitogenomic genes are not expressed in the mycelial stage but highly expressed in the sclerotium initial or developmental stage. These results indicate that some of mitochondrial genes may play a role in the development of sclerotium in *W. cocos*, which needs to be further elucidated in future studies. This study will stimulate new ideas on cytoplasmic inheritance of *W. cocos* and facilitate the research on the role of mitochondria in large sclerotium formation.

## Introduction

*Wolfiporia cocos* (Schwein.) Ryvarden & Gilb., a basidiomycete fungus that can form large sclerotia (Fuling in Chinese) close to the roots of conifers, such as *Picea*, *Tsuga*, and *Pinus*, is widely distributed in East Asia, Australia, North America, and Africa (Weber, 1929; Davidson and Campbell, 1954). The dried sclerotia have been used as a sedative, stomachic, and diuretic in Chinese and Japanese herbal medicine for thousands of years (Wang et al., 2013). For example, Guizhi Fuling Capsule (also called Guizhi Fuling Wan) has been used as a traditional Chinese remedy to treat gynecological inflammatory diseases, including uterine fibroids, endometriosis, and primary dysmenorrhea (Hu et al., 2014). Additionally, *W. cocos* has been popularly added to nutraceuticals, tea supplements, wine supplements, cosmetics, and functional foods to improve their quality all over the world (Wang and Song, 2007; Zhang C. L. et al., 2015).

*Wolfiporia cocos* forms larger sclerotium than any other known sclerotium-forming fungi. In China, artificial cultivation of *W. cocos* can produce fresh sclerotia with a mean weight of 3–4 kg (Xu et al., 2014), while wild fresh sclerotia sometimes can reach more than a 100 kg. Up to now, little is known about why *W. cocos* could form such large sclerotia. In *Polyporus umbellatus*, sclerotial formation was triggered by low-temperature treatment, which was shown to enhance the reactive oxygen species (ROS) in mycelia and may be important in triggering sclerotial differentiation in this species (Xing et al., 2013). In several other sclerotium-forming fungi, oxidative stress from the increased ROS in cell was also demonstrated to be implicated in sclerotial formation (Georgiou et al., 2000; Georgiou and Petropoulou, 2001; Papapostolou and Georgiou, 2010; Papapostolou et al., 2014; Osato et al., 2017). *W. cocos* is a subterranean mushroom with a low concentration of oxygen, implying that oxidative stress may also play a role in sclerotial formation.

Endogenous ROS is mainly produced from mitochondria, and mitochondria are often referred to as “signaling hubs” within eukaryotic cells. Mitochondria, also called powerhouse, generate the universal cellular energy ATP through oxidative phosphorylation to support a diversity of cellular activities. They were reported to act as integrators of signals related to both plant development and stress response pathways (Liberatore et al., 2016). Meanwhile, mtDNA mutations may result in developmental disease in humans (Schon et al., 2012). Mitochondria are also reported to be important for the growth and development of fungi, participating in senescence, quiescence, virulence, pathogenicity, and drug resistance (Chatre and Ricchetti, 2014; Cárdenas-Monroy et al., 2017). Additionally, the mitogenome was also widely used to study the characterization of genetic variation, phylogenetic relationship, and evolution within a fungal population (Zhang et al., 2016; Zhang S. et al., 2017; Zhang Y. J. et al., 2017).

Up to now, 661 fungal mitogenomes are available in the NCBI Organelle Genome database^1^, accounting for 6% of the total published mitogenomes (10,610). Among them, 15 mitogenomes of 14 species in the order Polyporales have been included: *Fomitopsis palustris* (AP017926), *Trametes cingulata* (GU723273), *Trametes hirsuta* (NC_037239), *Phlebia radiata* (HE613568), *Laetiporus sulphureus* (MG519331), *Taiwanofungus camphoratus* (MH745717), *Ganoderma* sp. (MH252531), *Ganoderma lucidum* (MH2525322), *Ganoderma lucidum* (KC763799), *Ganoderma sinense* (NC_022933), *Ganoderma meredithae* (NC_026782), *Ganoderma applanatum* (NC_027188), *Ganoderma tsugae* (NC_037936), *Ganoderma leucocontextum* (NC_037937), and *Ganoderma calidophilum* (NC_037938) (Haridas and Gantt, 2010; Li et al., 2013, 2018, 2019; Salavirta et al., 2014; Wang et al., 2016a, b; Tanaka et al., 2017). Although the mitogenome of *W. cocos* PC1609, a cultivated strain in Korea was reported; it was reported briefly as an announcement without available sequence information (Lee et al., 2019).

In this study, the mitogenomes of two single-spore isolates (SSIs) from two widely geographically separated *W. cocos* heterokaryotic strains [the strain L, a cultivated strain for harvest of sclerotia in China (Xu et al., 2014) and strain MD-104 collected on *Pinus* fence post form Florida, Alachua, Gainesville, United States (Floudas et al., 2012)] were assembled based on the Illumina genome sequence, respectively. Characterizations of the two mitochondrial genomes in *W. cocos* were presented in detail first. Mitogenome comparison and phylogenetic analyses were performed to reveal their characteristics in variations, phylogenetic relationship, and evolution. To investigate whether mitochondrial genes are functional in the process of sclerotium development, the mitochondrial gene expressions in different sclerotium-developmental stages were analyzed.

## Materials and Methods

### Strains and DNA Extraction

Two SSIs MD-104 SS10 and BL16 with wide geographic separation were used in this study. The parental strain of MD-104 SS10 comes from Florida, United States, and it was kindly provided by Professor David Scott Hibbett (Clark University, United States). The parental strain of BL16 comes from China and is cultivated for harvest of sclerotia. The mycelia of the two SSIs were cultured on solid complete yeast medium (glucose 20 g L^–1^, yeast extracts 2 g L^–1^, peptone 2 g L^–1^, K_2_HPO_4_ 1 g L^–1^, MgSO_4_ 0.5 g L^–1^, KH_2_PO_4_ 0.46 g L^–1^, and agar 20 g L^–1^) at 25°C for five days. Finally, the mycelia were collected and DNA was extracted as described in the literature (Xiang et al., 2016).

### Sequencing, Assembly, and Sequence Verification

DNA samples of BL16 were sent to Beijing Biomarker Technologies (Beijing, China) for the whole-genome sequencing using an Illumina HiSeq 2500 Platform. The genome sequence of MD-104 SS10 was downloaded from the JGI database^2^. FastQC v0.11.8 was used to check the quality of read data, then Trimmomatic 0.33 was used to trim reads (nucleotides more than 50 in length with a quality score higher than 30 were retained) (Bolger et al., 2014). The mitogenome was assembled following the methods of Deng et al. (2018). Briefly, the obtained clean reads were assembled using SPA 3.8.1 (Bankevich et al., 2012). Mitochondrial related contigs were identified using local BLASTX against a database of known mitochondrial proteins from closely related species. The contigs were assembled into a finished circle molecule by manual overlap. Gap filling was performed using paired-end read relationships with MITObim 1.9 (Hahn et al., 2013), and finally, the assembled sequences were manually examined for errors. The mitogenomes were verified by PCR. Primer pairs are listed in Supplementary Table S1. The PCR reaction system (20 μL) consisted of 1 μL DNA, 0.5 μL forward/reverse primers, 10 μL 2 × Taq Master Mix (Vazyme, Nanjing), and 8 μL ddH_2_O. The amplification was performed with an initial denaturation at 95°C for 5 min, followed by 35 cycles of denaturation at 95°C for 30 s, annealing at 55 ± 3°C for 30 s, and extension at 72°C for × min (1 kb/1 min), then 72°C for 10 min and 12°C for 10 min. Finally, the amplified PCR products were subjected to electrophoresis analysis by 1% agarose gel and the gels were stained with ethidium bromide.

### Gene Annotation and Bioinformatics Analysis

Using the Mold, Protozoan, and Coelenterate Mitochondrial Code and the Mycoplasma/Spiroplasma Code (NCBI translation Table 4) as genetic code, the mitogenomes of the two SSIs were annotated with Mfannot^3^ and RNAweasel^4^, respectively. The published mitogenomes of several fungi belonging to Polyporales were downloaded and then used as references to correct the internal stop codons of the predicted genes using the software Web Apollo (Lee et al., 2013). The two mitogenomes were annotated in the NCBI database as follows: Open Reading Frames (ORFs) were predicted using NCBI ORF Finder^5^, and protein-coding and rRNA genes were annotated by BLASTp and BLASTn queries against non-redundant NCBI databases. Then, the two annotation results were compared and manually examined for errors. The putative tRNA genes and their secondary structures were further identified using software tRNAscan-SE 2.0 with the Mold and Protozoan Mito mode (Chan and Lowe, 2019).

The ideogram of the complete mitogenome was drawn using OGDraw (version 1.3.1)^6^. The basic composition of the mitogenome was analyzed using Lasergene v7.1 in the mold mode (Kumar et al., 2016), and the AT skew and GC skew were calculated using the formula (A−T)/(A + T) and (G−C)/(G + C), respectively. Codon usage was calculated with Condon Usage of the Sequence Manipulation Suite (Stothard, 2000). Tandem repeat was predicted by Tandem Repeats Finder^7^ with E-value < 10^–5^, and forward repeat, reverse repeat, complementary repeat, and palindromic repeat were analyzed using REPuter with E-value < 10^–5^ (Kurtz et al., 2001). The resulting repeat sequences were visualized using Circoletto^8^. Horizontal gene transfer (HGT) between mitochondria and nucleus was evaluated by comparing the mitogenome and the nuclear genome using Local BLAST with E-value < 10^–5^. Collinearity analysis of the mitogenomes among the two SSIs and fourteen Polyporales species or eight sclerotium-forming fungi was performed by Mauve 2.4.0, respectively (Darling et al., 2011).

### Phylogenetic Analysis

The amino acid sequences of 14 core-conserved ORFs (*atp6*, *atp8*, *atp9*, *nad1-6*, *nad4L*, *cox1-3*, and *cob*) were used for phylogenetic analysis. Two SSIs and other 28 fungal species (14 species of Polyporales, six fungal species of Basidiomycetes, and eight sclerotium-forming fungal species) were used for test (Supplementary Table S2). All amino acid sequences were aligned using MAFFT, and the best model of phylogenetic analysis was chosen by ModelFinder in PhyloSuite v1.1.15 (Zhang D. et al., 2018; Zhang S. et al., 2018). Bayesian Inference phylogenies were inferred using MrBayes 3.2.6 (Ronquist et al., 2012) under Wag + I + G + F model (20,000 generations). Finally, iTOL^9^ was used for annotation and display. The genetic distance, synonymous substitution rate (Ks), and non-synonymous substitution rate (Ka) of the above 14 PCGs were calculated by MEGA 7.0 (Kimura 2-parameter model) and DnaSP v6.10.01 (Rozas et al., 2017).

### Field Cultivation, Transcriptome Analyses, and Quantitative Real-Time PCR

The parental strain of BL16 (*W. cocos* strain L) was used for field cultivation to collect samples of different sclerotial developmental stages: mycelia in the bark of pine log (MBPL), initial stage of sclerotial formation (ISSF), and developmental stage of sclerotial formation (DSSF). Field cultivation of sclerotia was performed according to the traditional cultivation method named “short-cut pine log pit-casing cultivation” (PC) as reported by Xu et al. (2014). A cultivated experiment was performed from May to November 2018. Samples were RNA sequenced on Illumina HiSeq 2500 Platform by Beijing Genomics Institute. Clean reads were trimmed using Trimmomatic-0.33 (Bolger et al., 2014) to filter adaptor sequences, contamination, and low-quality reads. Hisat2 (Kim et al., 2015) was used to map clean reads to both *W. cocos* BL16 mitochondrial genome and nuclear genome, and the clean reads that were mapped to mitogenome but not to nuclear genome were selected for further analysis. The mapped clean read count was evaluated by HTSeq (Anders et al., 2015) and converted to Transcripts Per Million (TPM) data through TBtools (Chen et al., 2018).

The above results were verified by quantitative real-time PCR (qRT-PCR). Briefly, total RNA was extracted using RNAiso Plus Total RNA Extraction Kit (TaKaRa, Dalian, China) according to the manufacturer’s instructions. cDNAs were synthesized using HiScript II Q RT SuperMix for qPCR (+gDNA wiper) (Vazyme, Beijing, China). Using *PP2A* as an internal reference, qPCR was conducted in a 10-μL total volume containing 5 μL of AceQ qPCR SYBR Master SYBR (Vazyme, Nanjing, China), 1 μL of five times diluted cDNA, 0.5 μL of the forward and reverse primers (10 mM), and 3 μL of nuclease-free water. The thermal cycling parameters of the reactions were as follows: 95°C for 5 min, followed by 40 cycles of denaturation at 95°C for 10 s, 60°C for 30 s, and 95°C for 10 s, and the temperature of the melting curve was increased from 60 to 95°C at an interval of 0.5°C with a hold of 5 s at each step. Finally, the results were analyzed using a CFX Connect Real-Time PCR system (BIO-RAD, United States). The primers used are listed in Supplementary Table S1.

## Results

### General Features

**FIGURE 1:**
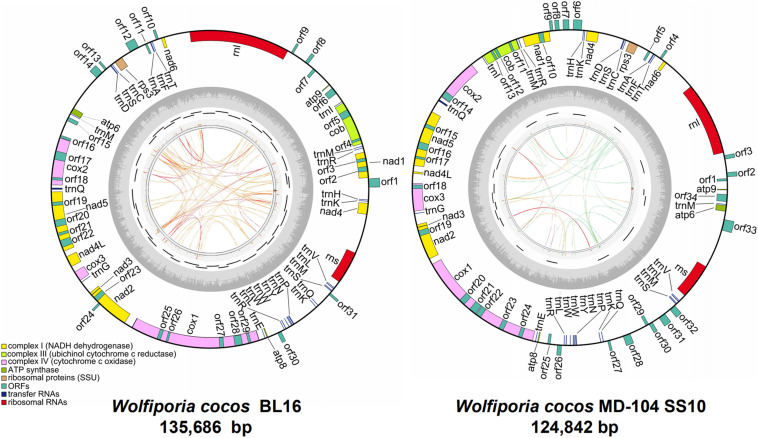
The ideogram of the two mitogenomes of *Wolfiporia cocos*. The outside ring with colorful blocks stands for mitochondrial ORFs, rRNAs, and tRNAs in *W. cocos*, and the blocks inside the ring stand for genes located on the J strand and the other side blocks stand for genes located on the N strand. The inside ring represents GC content. The inner side incomplete circle shows the PCR amplification regions. The innermost circle shows the repetitive sequences within mitogenomes, and the links of blue, green, orange, and red colors represent the score/max ratio, <0.25, <0.50, <0.75, and >0.75, respectively.

The mitogenomes of BL16 (MT079861) and MD-104 SS10 (MT079862) are typical circular DNA molecules of 135,686 bp and 124,842 bp (Figure 1), respectively. PCR amplification showed that the sequences of the assembled mitogenomes are reliable (Supplementary Figure S1). A total of 76 and 79 genes were identified in BL16 and MD-104 SS10, respectively (Supplementary Table S3). In BL16, 66 genes are located on the J strand, and 10 ORFs (*orf1*, *orf8-10*, *orf12-14*, *orf24*, *orf30*, and *orf31*) are located on the N strand (Figure 1). In MD-104 SS10, 65 genes are located on the J strand, and 14 ORFs (*orf2–4*, *orf6–9*, *orf26–28*, and *orf30–33*) are located on the N strand (Figure 1). The total GC content of the mitogenomes of BL16 and MD-104 SS10 are 34.9 and 34.8%, respectively. As commonly found in all the Polyporales mitogenomes examined, TTT (Phe), AAA (Lys), ATT (Ile), TTA (Leu), AAT (Asn), ATA (Ile), and TAT (Tyr) are the most frequently used codons in the two SSIs of *W. cocos* (Supplementary Table S4).

Meanwhile, 46 and 49 ORFs were identified in BL16 and MD-104 SS10, with a total size of 76,252 bp and 70,874 bp, accounting for 56.20 and 56.77% of the whole mitogenomes, respectively (Table 1). Among them, 14 are core-conserved ORFs typical to fungal mitogenomes (*nad1-6*, *nad4L*, *cox1-3*, *atp6*, *atp8*, *atp9*, and *cob*), which are reported to be associated with the mitochondrial inner membrane Complexes I, III, IV, and V of the respiratory chain, and one ribosomal protein (*rps3*) is related to self-replication. *cox1* and *atp9* are the largest and smallest core-conserved ORFs, which are 18,836 and 135 bp as well as 16,402 and 135 bp in BL16 and MD-104 SS10, respectively. BLAST analysis of these 15 ORFs revealed a very high sequence identity between the two SSIs (a significant hit of 96.86–100% in nucleotide sequence identity and 75.79–100% amino acid sequence identity) (Supplementary Table S3). The nad2/nad3 genes were joined by an overlap of one base pair in both the SSIs (Supplementary Table S3). The two SSIs vary in non-conserved ORFs (ncORFs), with 31 in BL16 while 34 in MD-104 SS10, including 14 free-standing ncORFs (located in the intergenic regions) and 17 intronic ncORFs (located in the intronic regions) in BL16, and 19 free-standing ncORFs and 15 intronic ncORFs in MD-104 SS10 (Supplementary Table S3). Some of the ncORFs may be functional because they contain LAGLIDADG or GIY-YIG or group I intron conservative domain or encode proteins similar to homing RNA or DNA polymerase, while the functions of 15 ncORFs in BL16 and 16 ncORFs in MD-104 SS10 are unknown (Supplementary Table S3). A comparison of the sequence similarity of the ncORFs showed that above 50% ncORFs are conserved in the two *W. cocos* SSIs, with a significant hit (E-value < E^–20^) and high sequence identity between 17 ncORFs in BL16 and 18 ones in MD-104 SS10 (Supplementary Table S3).

**TABLE 1:** Gene features of the two mitogenomes of *Wolfiporia cocos*.

Mitogenome composition	Items	Strains	
		BL16	MD-104 SS10
Protein-coding region	Number of ORFs	46	49
	Size of total of ORFs (bp) (%)	76,252 (56.20)	70,874 (56.77)
	Free-standing ORFs (bp) (%)	65,329 (48.15)	61,712 (49.43)
	Intronic ORFs (bp) (%)	10,923 (8.05)	9,162 (7.34)
Intron region	Number of introns	26	25
	Intronic region (bp) (%)	43,685 (32.20)	35,907 (28.76)
RNA region	Number of rRNAs	2	2
	Number of tRNAs	28	28
	rRNA regions (bp) (%)	18,427 (13.58)	13,920 (11.15)
	tRNA regions (bp) (%)	2,250 (1.66)	2,243 (1.80)
Intergenetic region	Intergenetic regions (bp) (%)	48,501 (35.75)	46,967 (37.62)
Repeated region	Repeated region (bp) (%)	14,229 (10.49)	5,619 (4.5)
	Horizontal gene transfer region (bp) (%)	2,807 (2.07)	628 (0.50)

*The symbol % means accounting for the percentage of the whole mitogenome.*

**FIGURE 2:**
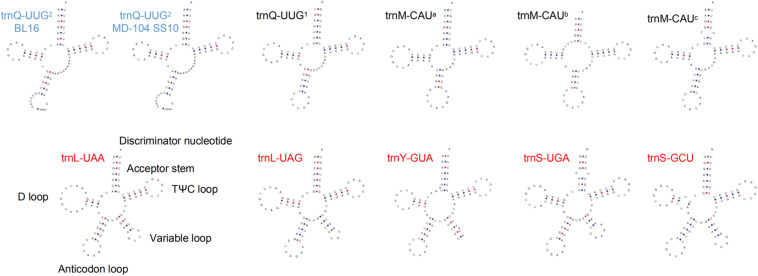
Some tRNA putative secondary structures of *Wolfiporia cocos*. Five tRNAs (trnS-GCU, trnL-UAA, trnY-GUA, trnS-UGA, and trnL-UAG) with red letters represent tRNAs with a variable loop. Two tRNAs with blue letters stand for tRNAs with an intron. trnM-CAU^a^, trnM-CAU^b^, and trnM-CAU^c^ stand for three different trnM sequences with the same anticodon; trnQ-UUG^1^ and trnQ-UUG^2^ represent two different trnQ sequences with the same anticodon.

Both the two mitogenomes contain 28 tRNA genes and two rRNA genes, with a total size of 2,250 bp and 18,427 bp in BL16 while 2243 bp and 13,920 bp in MD-104 SS10, accounting for 1.66 and 13.58% as well as 1.80 and 11.15% of the whole mitogenomes, respectively (Table 1). The tRNAs encode 20 standard amino acids and vary in size from 71 to 85 bp except for trnQ-UUG^2^, which is 236 and 229 bp and contains an intron with a size of 160 and 153 bp in BL16 and MD-104 SS10, respectively (Supplementary Table S3). To our knowledge, this is the first report about the presence of intron in tRNA in fungi. Two rRNA genes and 28 tRNA genes also showed a very high sequence identity between the two SSIs, with 100% sequence identity in the majority of genes except for *rnl*, *rns*, trnL-UAA, trnL-UAG, and trnQ-UUG^2^ (Supplementary Table S3). tRNAs can be folded into a classical cloverleaf secondary structure, and some typical tRNAs are shown in Figure 2. In the two SSIs, the variable loop is found in trnS-GCU, trnL-UAA, trnY-GUA, trnS-UGA, and trnL-UAG, and a total of 45 single GU mismatches are identified. Two tRNA genes with different anticodons encode lysine, leucine, arginine, serine, and tryptophan; methionine and glutamine are encoded by three and two tRNAs with the same anticodons, respectively.

### Intron Variation

**FIGURE 3:**
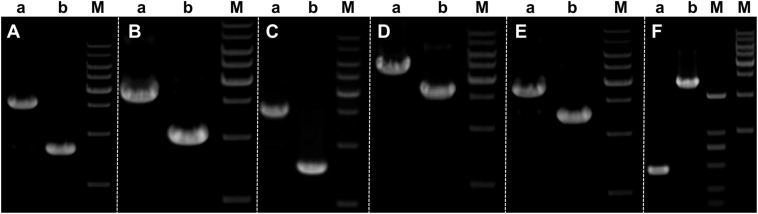
Validation of intron differences in size in the two mitogenomes of *Wolfiporia cocos*. Primers designed to validate the differences in introns were as follows: **(A)** *nad2*-i2; **(B)** *nad5*-i1; **(C)** *cob*-i2; **(D)** *cox1*-i1; **(E)** *cox2*-i3; and **(F)** *cox3*-i2. Lane a represents *W. cocos* SSI BL16, and lane b represents *W. cocos* SSI MD-104 SS10. M stands for the 1-kb marker (from up to down: 10,000, 8000, 6000, 5000, 4000, 3000, 2000, and 1000 bp) except for the second (from left to right) M which stands for BM2000 Marker (from up to down: 2000, 1000, 750, 500, 250, and 100 bp), respectively. The gels of different batches were separated by the white dotted line.

There are 26 introns in BL16 and 25 in MD-104 SS10 with the size of 43,685 and 35,907 bp, accounting for 32.20 and 28.76%, respectively (Table 1). Introns are found in eight core conserved ORFs (*nad1*, *nad2*, *nad4L*, *nad5*, *cox1*, *cox2*, *cox3*, and *cob*) and trnQ-UUG^2^ in the two SSIs, and the most introns are found in *cox1* (eight in BL16 and nine in MD-104 SS10). Except for *nad2*-i1 and trnQ-UUG^2^, introns are identified in group I (mainly in group IB), and two or more types of introns are identified in some genes, such as six intron types in *cox1*-i5 in BL16 and five types in *cox1*-i6 in MD-104 (Supplementary Table S5). The number and size of introns within the same gene are different in the two SSIs. For example, eight introns in *cox1* are identified in BL16 while nine in MD-104 SS10; obvious size differences are found between BL16 and MD-104 SS10 in the introns of *nad2*-i2, *cob*-i2, *cox1*-i1, *cox1*-i6, *cox2*-i3, and *cox3*-i2 (Supplementary Table S5). To verify the differences of introns between the two SSIs, six of the above nine introns were randomly selected for PCR. The results showed that all designed primer pairs could be used to discriminate the two SSIs (Figure 3), suggesting their potential to identify different strains by determination of the mitochondrial genotypes of *W. cocos* strains.

### Repeat Elements

In some fungal species, repetitive elements are reported to contribute to mitogenome expansion (Losada et al., 2014; Li et al., 2018; Liu et al., 2020). BLASTn searches of the *W. cocos* mitogenome against itself revealed 260 repeat regions in BL16 and 112 ones in MD-104 SS10, with a total length of 14,229 and 5619 bp, accounting for 10.5 and 4.5% of the whole mitogenome, respectively (Table 1). The size of the repeats ranged from 28 to 325 bp in BL16 with a repeat sequence similarity between 78.77 and 100%, and the size of the repeats ranged from 28 to 160 bp in MD-104 SS10 with a repeat sequence similarity between 82.31 and 100%. The largest repeat region in both BL16 and MD-104 SS10 is located in the intergenic region between *rnl* and *atp9*-*atp9*/*orf7* (*orf1* in MD-104 SS10) (Supplementary Table S6). In the mitogenomes of BL16 and MD-104 SS10, 28 and 19 tandem repeats are detected in a size range from 5 to 28 bp and 5 to 45 bp, respectively (Supplementary Table S7). The copy number is between two and nine, and most tandem repeats have two copies. We identified 151 forward, 10 reverse, 4 complementary, and 61 palindrome repeats (E-value < 10^–5^) in BL16, versus 75, 7, 0, and 32 in MD-104 SS10 (Supplementary Table S8), indicating more repeat elements in BL16 than in MD-104 SS10.

To identify that gene segments had been transferred between the nuclear and mitochondrial genomes, we blasted nuclear genomic sequences against the respective mitogenome. Data showed 24 and 11 aligned fragments, 39–427 bp in BL16 and 35–143 bp in MD-104 SS10, with a sequence identity between 86 and 100% (Supplementary Table S9). The largest aligned fragment was found to encompass the protein coding region *rns* in BL16 and *cox1*-i8 in MD-104 SS10, respectively (Supplementary Table S9).

### Gene Rearrangements and Mitogenome Synteny

**FIGURE 4:**
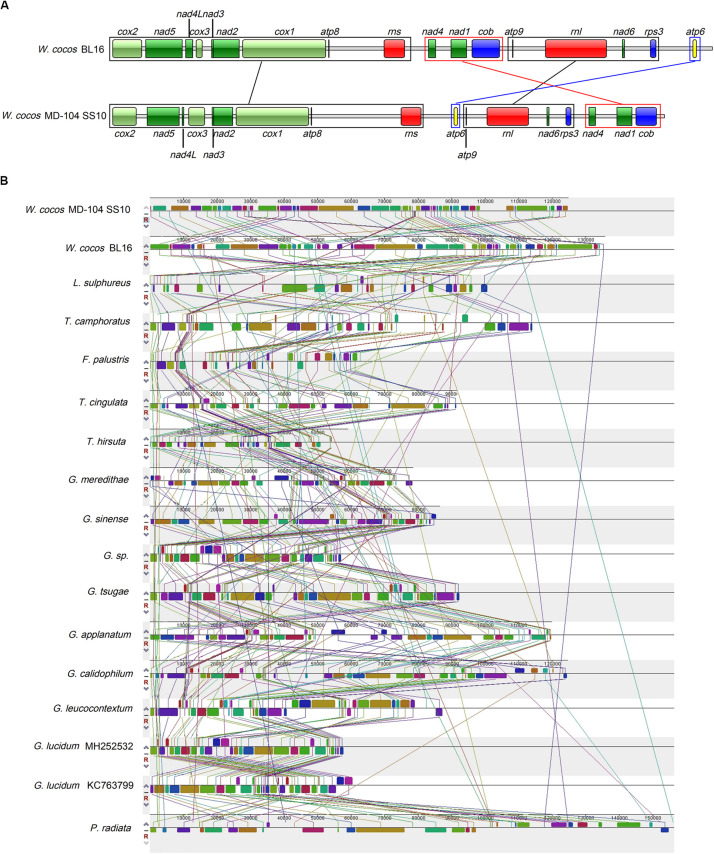
Gene order analysis in *W. cocos* and mitogenome synteny in the order Polyporales. **(A)** Gene order in the two mitogenomes of *W. cocos*. Fourteen core-conserved protein coding genes, *rps3*, *rnl*, and *rns* were used in the gene order analysis. The black blocks and lines show the genes with the same order in the two mitogenomes; the red block and lines stand for rearrangement of the gene block “*nad4*-*nad1*-*cob*”; the blue block and lines represent rearrangement of *atp6.* **(B)** Collinearity analysis of the mitogenomes in the order Polyporales.

The gene orders of the 14 conserved ORFs as well as *rps3*, *rnl*, and *rns* genes across these two *W. cocos* SSIs and other 14 Polyporales fungi were investigated. Based on relative gene order, *W. cocos* might have a closer relationship with *L. sulphureus* (Supplementary Figure S2). Except for five *Ganoderma* species and two *Trametes* species, variations are observed in the gene orders of above mentioned conserved genes across 15 Polyporales species (Supplementary Figure S2). The most salient feature is an obvious gene rearrangement between the two *W. cocos* SSIs. As shown in Figure 4A, the gene orders of the region *nad4-cob* and *atp6* are reversed, with the region *nad4-cob* being located in front of and behind the region *atp9-rps3* in BL16 and MD-104 SS10, respectively, and it is opposite for *atp6*.

Mitogenome synteny analysis indicated the occurrence of many gene rearrangements in the mitogenomes across Polyporales species (Figure 4B). There are a total of 55 homologous fragments across 17 mitogenomes in 15 Polyporales fungi, and these fragments varied in number, size, and relative position (Figure 4B). *G. Calidophilum*, and *G. lucidum* are shown to have the most (55) and the least (37) homologous fragments, while 50 and 47 homologous fragments are detected in *W. cocos* MD-104 SS10 and *W. cocos* BL16. The number of homologous fragments does not seem to be relevant to the size of the tested Polyporales mitogenomes. For example, *P. radiata* (156,348 bp) with 46 homologous fragments is larger than *G. leucocontextum* (88,194 bp) with 52 homologous fragments. Additionally, despite a relative high degree of synteny between the *Ganoderma* species and two *Trametes* species, obvious gene rearrangements are shown across the tested Polyporales mitogenomes.

**FIGURE 5:**
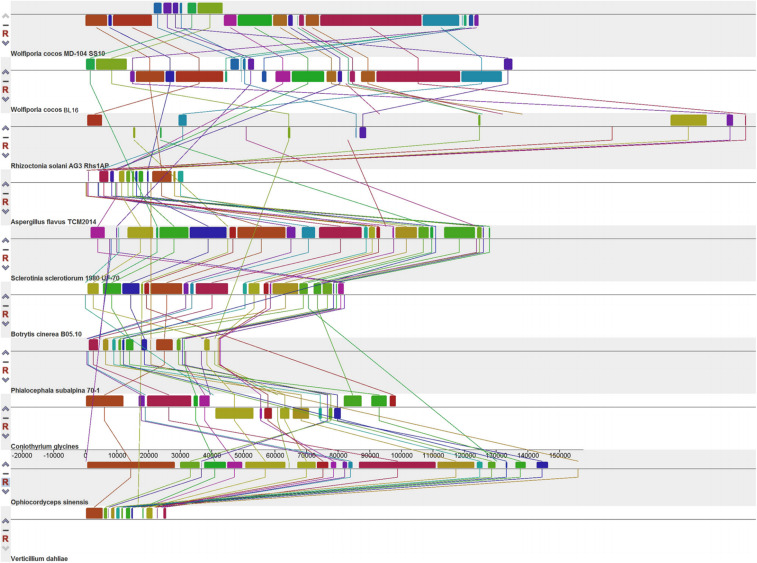
Collinearity analysis of the 10 mitogenomes of sclerotium-forming fungi.

To investigate the possible clues associated with sclerotial formation from the mitogenomes, the mitogenome synteny of ten sclerotium-forming fungi, including the two *W. cocos* SSIs, one Basidiomycetes fungus *Rhizoctonia solani*, and seven Ascomycetes fungi (Figure 5), was analyzed. A total of 29 homologous fragments and three common ones are found among these tested mitogenomes. The three common homologous fragments cover *cox1*, *orf31*-*trnS*-*trnM*-*trnL*-*trnV*-*rns*, and *orf6* in BL16 and MD-104 SS10, respectively.

### Phylogenetic Analysis

**FIGURE 6:**
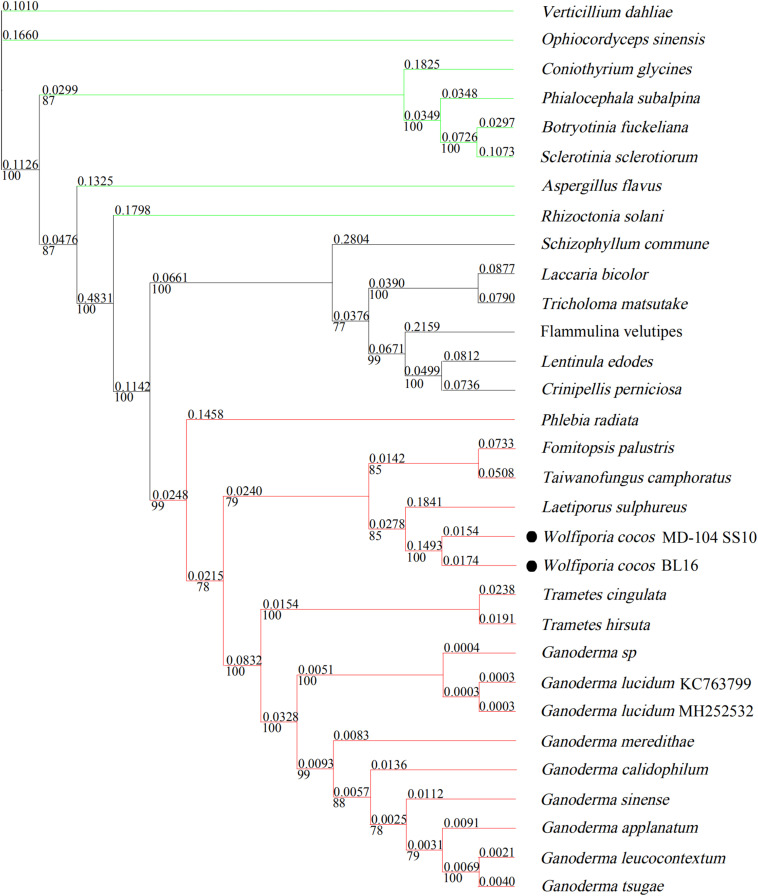
Molecular phylogenetic tree of 31 mitogenomes based on Bayesian inference of the 14 core PCGs. The numbers above and beneath the branch stand for evolutionary distance and bootstrap evaluation. The green line represents sclerotium-forming fungi; the red line represents 15 species belonging to Polyporales; the black line stands for species in the order Agaricales.

In Figure 6, seven tested Ascomycetes fungi were shown to be obviously separated from the other tested 24 Basidiomycetes fungi in the phylogenetic tree; all the tested Agaricales fungi and Polyporales fungi cluster together. *W. cocos* has a sister relationship with *L. sulphureus*, which is consistent with the results of the gene rearrangement analysis (Figure 4A). The genetic distance of 14 core conserved ORFs (*cox1*-*3*, *nad1*-*6*, *nad4L*, *atp6*, *atp8*, *atp9*, and *cob*) among the 17 mitogenomes in Polyporales was also analyzed, and the results showed that the genetic distance varies from 0.05 (*nad2*) to 0.17 (*cob*) based on the Kimura-2-parameter model (Supplementary Figure S3A). The Ka/Ks of these genes was below 1 and varied from 0.05 (*nad4L*) to 0.3 (*cob*) (Supplementary Figure S3B), indicating that the 14 core conserved ORFs were subjected to purification selection.

### Mitochondrial Gene Expressions in Different Sclerotium-Developmental Stages

**FIGURE 7:**
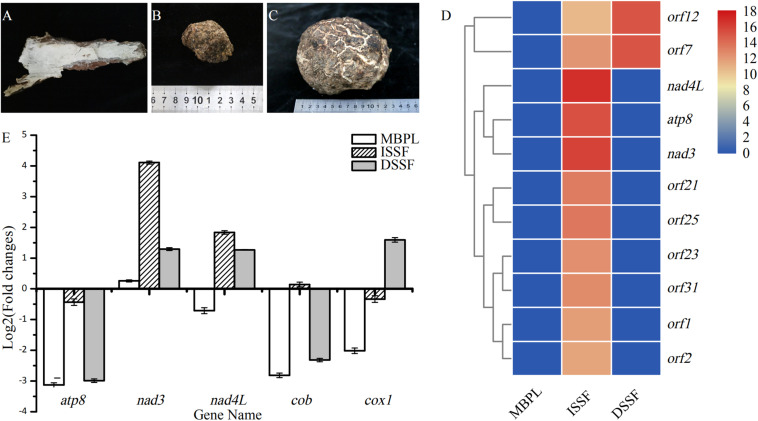
Mitochondrial gene expressions. **(A)** Mycelia in the bark of pine log (MBPL); **(B)** initial stage of sclerotial formation (ISSF); **(C)** developmental stage of sclerotial formation (DSSF); **(D)** the hierarchical clustering of 11 ORFs with a significant differential expression by transcriptome analysis in different sclerotium-developmental stages; **(E)** validation of expression levels of obviously differentially expressed gene by qRT-PCR, and the error bar denotes the standard deviation of the three qRT-PCR replicates.

Based on the transcriptome data, 37 out of the 46 ORFs in BL16 are expressed during the development of sclerotia, including 14 core conserved ORFs, *rps3*, and 22 ncORFs. The total TPM varied from 3036.748 (*orf2*, LAGLIDADG DNA endonuclease) to 1,040,684.451 (*orf9*, function unknown). Interestingly, 24 ORFs are not expressed in the MBPL (MBPL, Figure 7A) stage, while the majority of them are expressed in the ISSF (ISSF, Figure 7B) and DSSF (DSSF, Figure 7C) stage with a very high TPM (Supplementary Table S10), indicating that these ORFs might be related to sclerotium formation.

Three genes, *cox1*, *orf31*, and *orf6*, are revealed as three common homologous genes identified across 10 sclerotium-forming fungi by above homologous fragment analysis, and they are also significantly differentially expressed during sclerotial formation (Supplementary Table S10). *Cox1* showed high expression in all the three sclerotium-developmental stages and was significantly upregulated in the ISSF stage compared to the MBPL and DSSF stages (Supplementary Table S10); *Orf31* is only expressed in the ISSF stage, and *orf6* is only expressed in the DSSF stage (Supplementary Table S10). Additionally, seven conserved ORFs (*atp6*, *atp8*, *atp9*, *cox2*, *nad3*, *nad4L*, and *nad6*) and 12 ncORFs (*orf1–3*, *orf7*, *orf8*, *orf12*, *orf17*, *orf20–23*, *orf25*, and *orf31*) are obviously upregulated in the ISSF stage when compared with their expression levels in the MBPL stage. Five ncORFs (*orf4*, *orf7*, *orf11*, *orf12*, and *orf14*) are obviously upregulated in the DSSF stage as compared with their expression levels in the ISSF stage. Five conserved ORFs (*nad3*, *nad4L*, *atp8*, *nad4*, and *nad5*) and nine ncORFs (*orf1*, *orf2*, *orf19*, *orf21*, *orfr23*, *orf25*, *orf28*, *orf30*, and *orf31*) are obviously downregulated in the DSSF stage versus their expressions in the ISSF stage (Supplementary Table 10).

The hierarchical clustering of 11 ORFs with significant differential expressions across the three tested stages is shown in Figure 7D. The ncORFs “*orf12* and *orf7*” are clustered together, indicating a gradual upregulation in their expression from the MBPL stage to the DSSF stage, and the other nine ORFs exhibit an upregulated expression in the MBPL stage and a downregulated expression in the DSSF stage. Additionally, the functional-related genes, such as “*nad3*, *nad4L*, and *atp8*” (respiratory chain complexes) and “*orf2* and *orf23*” (LAGLIDADG DNA endonuclease), are clustered together. The expression of several of the aforementioned genes was further verified by qRT-PCR, and the five tested genes were shown to have the same expression pattern as that in the transcriptome analysis (Figure 7E).

## Discussion

The reported mitogenomes in Polyporales varied from 57,232 to 156,348 bp in size. In this study, we firstly systematically analyzed the mitogenomes of the two *W. cocos* SSIs with a size of 135,686 bp (BL16) and 124,842 bp (MD-104 SS10), which, to our knowledge, are the second and third largest mitogenomes after *P. radiata* (156,348 bp) among Polyporales fungi, respectively (Salavirta et al., 2014). Previous studies have shown that the number and sequence length of intron and ncORFs, intergenic region, and direct introduction of exogenous DNA (such as plasmid-related DNA and sequence duplication) may contribute to the expansion of the fungal mitogenomes (Salavirta et al., 2014; Li et al., 2018, 2019; Liu et al., 2020). Here, the comparison of the two mitogenomes also revealed that the length of *W. cocos* mitogenome seems to be affected by the lengths of some regions, including protein-coding region, intronic and intergenic regions, and repeated and HGT regions (Table 1). The lengths of rRNAs were seldom reported to vary previously. In the present study, *W. cocos* isolates have two rRNAs of different sizes, which contribute to enlarge the size of *W. cocos* mitogenome (Table 1). Despite the size variation between the two tested *W. cocos* isolates, their mitogenome sequences showed high sequence identity in 14 core-conserved ORFs, *rps3*, tRNAs, and about half of the ncORFs (Supplementary Table S3). ncORFs are reported to be poorly conserved even among closely related species (Li et al., 2015). About half of the ncORFs showed high sequence identity between the two isolates with wide geographic separation, indicating they are conserved in *W. cocos* and developed within species over time.

Generally, introns tend to be found typically in the conserved mitochondrial genes and occasionally in rRNAs. For example, a total of 34 introns were identified in seven typical conserved mitochondrial genes and two rRNAs of *Morchella importuna* (Liu et al., 2020). Up to now, there is no intron identified in tRNA in fungi. Here, we identified an intron for the first time in trnQ-UUG^2^ in both the mitogenomes of BL16 (160 bp) and MD-104 SS10 (153 bp). Several studies revealed possible functions of the intron, such as its potential involvement in the regulation of gene or alternative splicing (Thatcher et al., 2014; Shaul, 2017). Whether the intron in trnQ-UUG^2^ is functional needs further research. Mitogenome sequences, especially 14 core-conserved ORFs, were often used in phylogenetic relationship and evolution (Zhang Y. J. et al., 2015; Choudhary et al., 2018; Li et al., 2019). Here, the genetic distance of 14 core-conserved ORFs in *W. cocos* varied from 0.05 (*nad2*) to 0.17 (*cob*) based on the Kimura-2-parameter model, and the result of Ka/Ks indicated the purification selection of these genes. As reported by Li et al. (2018), *F. palustris* has a sister relationship with *L. sulphureus.* In this study, *F. palustris* has the closest relationship with *T. camphoratus*, indicating *L. sulphureus* is closer to *W. cocos* than *F. palustris*. This result could contribute to our understanding of evolution relationship among the fungi of Polyporales.

Sclerotial development is important for *W. cocos* production. The major problem currently facing the growers of *W. cocos* is the instability of production partially for little information known in sclerotium formation. In this study, *cox1*, *orf31*, and *orf6* are revealed as three common homologous genes identified across 10 sclerotium-forming fungi by homologous fragment analysis, and they are also significantly differentially expressed during sclerotial formation (Supplementary Table S10). These results indicate that they are functional in sclerotium development, but at a different phase. Their true function in *W. cocos* needs further research. Additionally, 22 of the 31 ncORFs are expressed in at least one of the three sclerotium-developmental stages. Until now, the roles of ncORFs remain a mystery, and recently, ncORFs have been reported to work possibly at least in the vegetative growth stage of *M. importuna* (Liu et al., 2020). Here, most of ncORFs also seem to be functional in the initial and developmental stages of sclerotium formation, with the highest expression in the initial stage in *W. cocos*. These results may broaden our knowledge that mitochondrion is implicated in large sclerotium formation in *W. cocos*, but it needs to be further elucidated. As previously reported, the increased ROS in cell is implicated in sclerotial formation (Georgiou et al., 2000; Georgiou and Petropoulou, 2001; Papapostolou and Georgiou, 2010; Xing et al., 2013; Papapostolou et al., 2014; Osato et al., 2017). The endogenous ROS is mainly produced from mitochondria, and whether some of the mitochondrial genes mentioned above are involved in the production of ROS, or how they work in the sclerotial formation in *W. cocos*, needs to be further explored.

## Data Availability Statement

The datasets generated for this study can be found in the NCBI, MT079861 and MT079862.

## Author Contributions

ZX and YB conceived and designed the experiments. YD assembled the draft mitochondrial genomes. NC and TW finished the field cultivation. MC performed the data analysis and experimental verification. ZX and MC wrote the manuscript. All authors contributed to the article and approved the submitted version.

## Conflict of Interest

The authors declare that the research was conducted in the absence of any commercial or financial relationships that could be construed as a potential conflict of interest.

## Footnotes

1 https://www.ncbi.nlm.nih.gov/genome/browse#!/organelles/

2 https://genome.jgi.doe.gov/Wolco1/Wolco1.home.html

3 http://megasun.bch.umontreal.ca/cgi-bin/mfannot/mfannotInterface.pl

4 http://megasun.bch.umontreal.ca/cgi-bin/RNAweasel/

5 https://www.ncbi.nlm.nih.gov/orffinder/

6 https://chlorobox.mpimp-golm.mpg.de/OGDraw.html

7 http://tandem.bu.edu/trf/trf.advanced.submit.html

8 http://tools.bat.infspire.org/circoletto/

9 https://itol.embl.de/tree/

## Supplementary Material

FIGURE S1 Validation of the two assembled mitogenomes of *Wolfiporia cocos.* **(A)** Validation of BL16 assembled mitogenomes; M, 1 kb Marker; 1–36, Primer pairs WcB16-1–36; **(B)** Validation of MD-104 SS10 assembled mitogenomes; M, BM2000 (except for the last one for BM2000 Marker); 1–18, Primer pairs WcSS10-1–18.

FIGURE S2 Gene order in the 17 mitogenomes in the order Polyporales. Fourteen core conserved protein coding genes, *rps3*, *rnl*, and *rns* were used in the gene order analysis. Genes conserved across at least 10 mitogenomes are shown in red; genes conserved across 7–10 mitogenomes are shown in firebrick; genes conserved across two or three mitogenomes are shown in light pink; others are shown in mistyrose.

FIGURE S3 Genetic analysis of 14 core conserved PCGs in the 17 Polyporales mitochondrial genomes. K2P, the Kimura-2-parameter distance; Ks, the number of (synonymous) substitutions per synonymous site; Ka, the number of (non-synonymous) substitutions per non-synonymous site.
