# Supplementary figures and images for: Characterization of Two Mitochondrial Genomes and Gene Expression Analysis Reveal Clues for Variations, Evolution, and Large-Sclerotium Formation in Medical Fungus Wolfiporia cocos
Source: Front Microbiol. 2020 Aug 4;11:1804. doi: 10.3389/fmicb.2020.01804 (PMC7417453; doi:10.3389/fmicb.2020.01804)

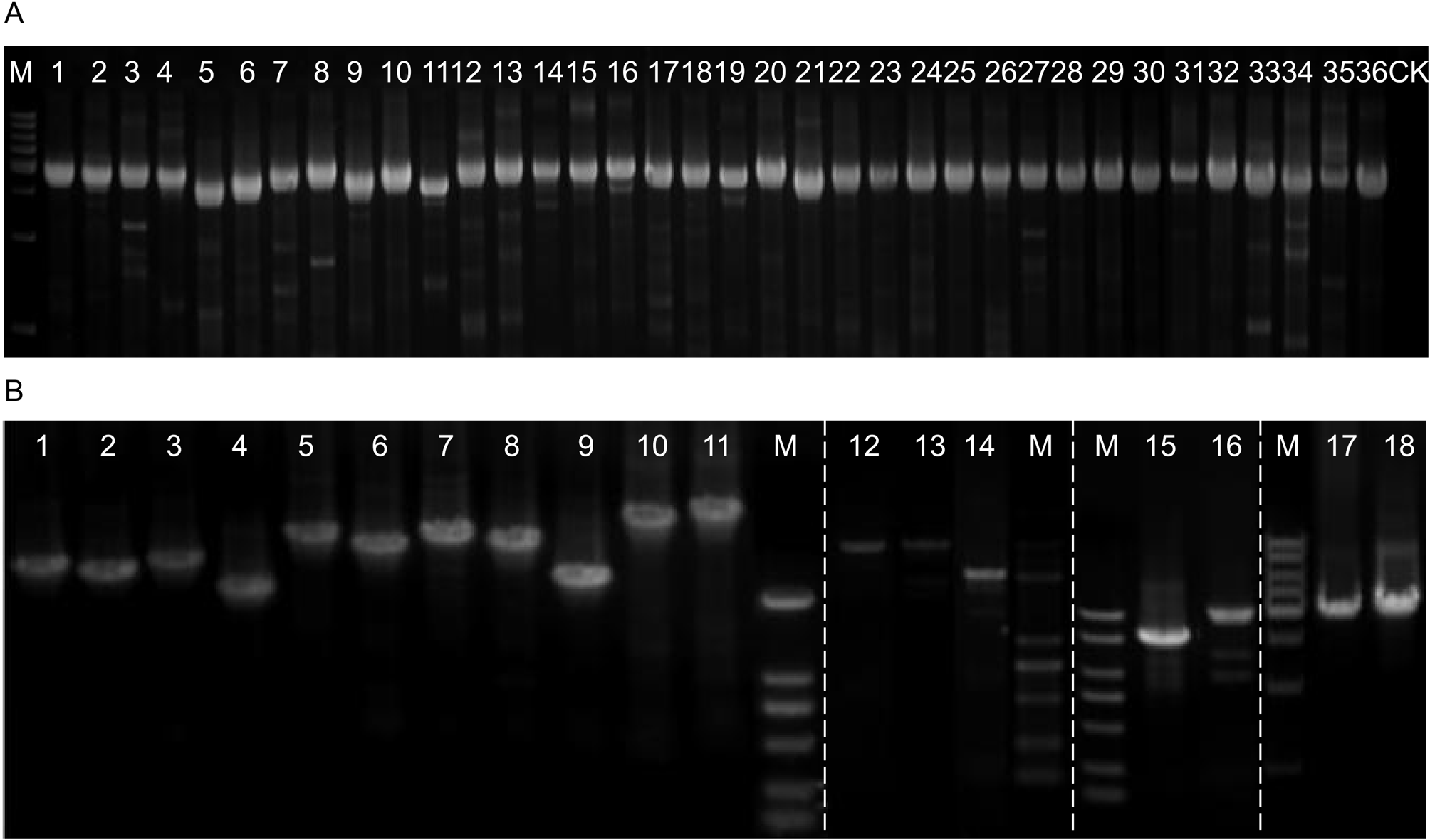

Supplement: FIGURE S1 — Validation of the two assembled mitogenomes of Wolfiporia cocos. (A) Validation of BL16 assembled mitogenomes; M, 1 kb Marker; 1–36, Primer pairs WcB16-1–36; (B) Validation of MD-104 SS10 assembled mitogenomes; M, BM2000 (except for the last one for BM2000 Marker); 1–18, Primer pairs WcSS10-1–18. [file Image_1.TIF]

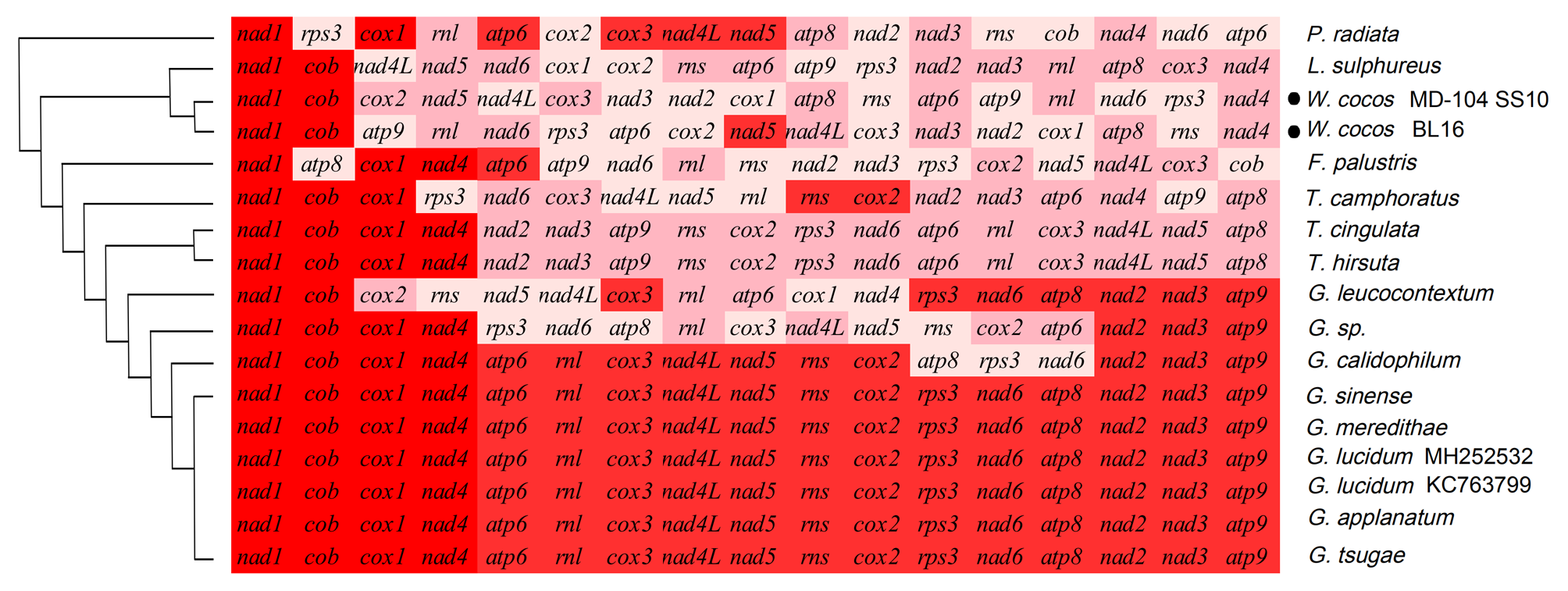

Supplement: FIGURE S2 — Gene order in the 17 mitogenomes in the order Polyporales. Fourteen core conserved protein coding genes, rps3, rnl, and rns were used in the gene order analysis. Genes conserved across at least 10 mitogenomes are shown in red; genes conserved across 7–10 mitogenomes are shown in firebrick; genes conserved across two or three mitogenomes are shown in light pink; others are shown in mistyrose. [file Image_2.TIF]

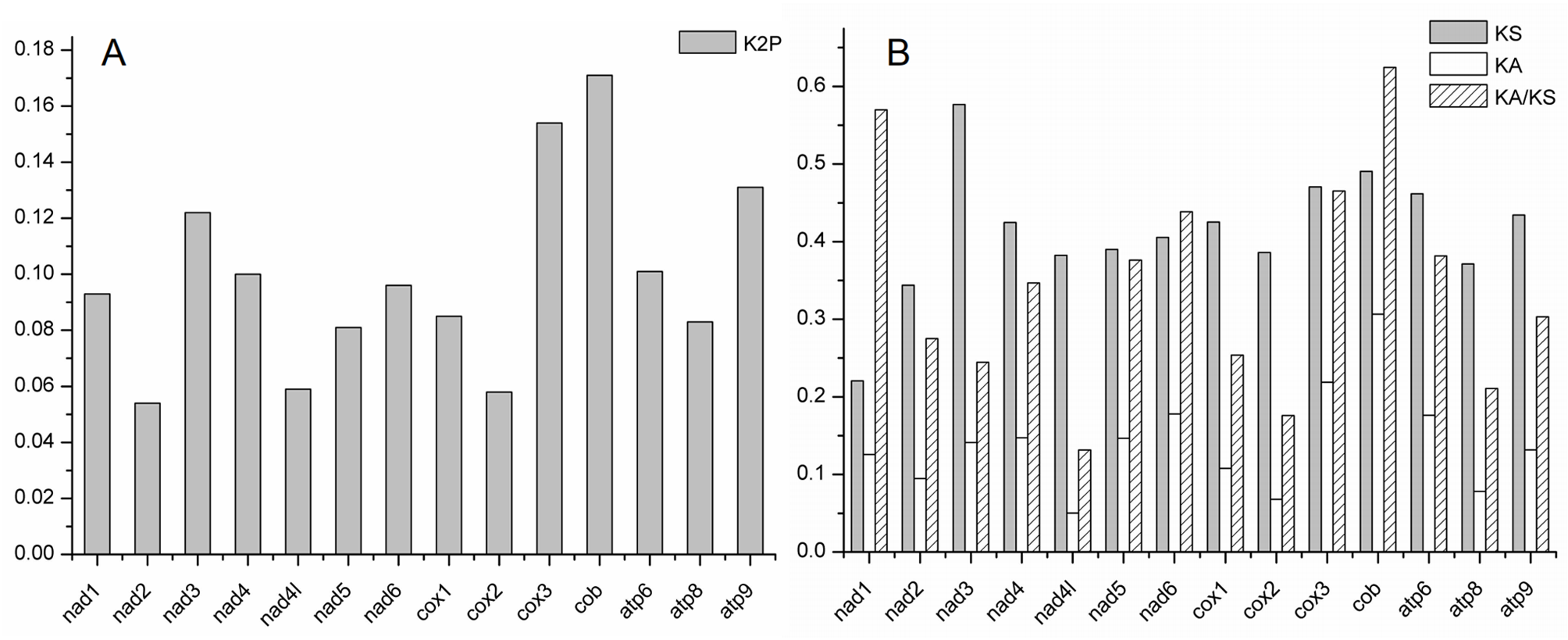

Supplement: FIGURE S3 — Genetic analysis of 14 core conserved PCGs in the 17 Polyporales mitochondrial genomes. K2P, the Kimura-2-parameter distance; Ks, the number of (synonymous) substitutions per synonymous site; Ka, the number of (non-synonymous) substitutions per non-synonymous site. [file Image_3.TIF]
